# Assessing the Bacterial Community Composition of Bivalve Mollusks Collected in Aquaculture Farms and Respective Susceptibility to Antibiotics

**DOI:** 10.3390/antibiotics10091135

**Published:** 2021-09-20

**Authors:** Vanessa Salgueiro, Lígia Reis, Eugénia Ferreira, Maria João Botelho, Vera Manageiro, Manuela Caniça

**Affiliations:** 1 National Reference Laboratory of Antibiotic Resistances and Healthcare Associated Infections, Department of Infectious Diseases, National Institute of Health Dr. Ricardo Jorge, 1649-016 Lisbon, Portugal; vanessa.salgueiro@insa.min-saude.pt (V.S.); ligia.reis@insa.min-saude.pt (L.R.); Eugenia.Ferreira@insa.min-saude.pt (E.F.); vera.manageiro@insa.min-saude.pt (V.M.); 2 Centre for the Studies of Animal Science, Institute of Agrarian and Agri-Food Sciences and Technologies, University of Porto, 4051-401 Porto, Portugal; 3 Division of Oceanography and Marine Environment, Portuguese Institute for the Sea and Atmosphere, 1749-077 Lisbon, Portugal; mjbotelho@ipma.pt; 4 CIIMAR, Interdisciplinary Centre of Marine and Environmental Research, University of Porto, 4450-208 Matosinhos, Portugal; 5 CIISA, Center for Interdisciplinary Research in Animal Health, Faculty of Veterinary Medicine, University of Lisbon, 1300-477 Lisbon, Portugal

**Keywords:** bivalve mollusks, aquaculture, antibiotic resistance, oxytetracycline, PMQR

## Abstract

Aquaculture is a growing sector, providing several products for human consumption, and it is therefore important to guarantee its quality and safety. This study aimed to contribute to the knowledge of bacterial composition of *Crassostrea gigas*, *Mytilus* spp. and *Ruditapes decussatus*, and the antibiotic resistances/resistance genes present in aquaculture environments. Two hundred and twenty-two bacterial strains were recovered from all bivalve mollusks samples belonging to the *Aeromonadaceae*, *Bacillaceae*, *Comamonadaceae*, *Enterobacteriaceae*, *Enterococcaceae*, *Micrococcaceae*, *Moraxellaceae*, *Morganellaceae*, *Pseudomonadaceae*, *Shewanellaceae*, *Staphylococcaceae*, *Streptococcaceae*, *Vibrionaceae*, and *Yersiniaceae* families. Decreased susceptibility to oxytetracycline prevails in all bivalve species, aquaculture farms and seasons. Decreased susceptibilities to amoxicillin, amoxicillin/clavulanic acid, cefotaxime, cefoxitin, ceftazidime, chloramphenicol, florfenicol, colistin, ciprofloxacin, flumequine, nalidixic acid and trimethoprim/sulfamethoxazole were also found. This study detected six *qnrA* genes among *Shewanella algae*, ten *qnrB* genes among *Citrobacter* spp. and *Escherichia coli*, three *oqxAB* genes from *Raoultella ornithinolytica* and *bla*_TEM-1_ in eight *E. coli* strains harboring a *qnrB19* gene. Our results suggest that the bacteria and antibiotic resistances/resistance genes present in bivalve mollusks depend on several factors, such as host species and respective life stage, bacterial family, farm’s location and season, and that is important to study each aquaculture farm individually to implement the most suitable measures to prevent outbreaks.

## 1. Introduction

Aquaculture is an ancient activity, practiced since the Roman Empire (140 B.C.) in Europe. It has developed over the centuries, but it was in the last three decades that it experienced its greatest growth, pressured by increased demand [1,2].

Bivalve mollusks are known to be rich in proteins, vitamin D, long-chain omega-3 fatty acids, iodine and selenium, contributing to a healthy diet [3]. These organisms represent the main aquaculture production in Portugal and, in 2012, 95.2% of the active establishments were for bivalve mollusks’ production [4]. In 2015, the main species produced were *Ruditapes decussatus*, with 2300 t, *Mytilus* spp., with 1200 t, and *Crassostrea gigas* and *Ostrea edulis*, with 650 t. Other important species are *Cerastoderma edule* (264 t). The central and southern regions of Portugal (regions B and A, respectively) are the most relevant in the national production of bivalve mollusks [4,5].

*C. gigas* (common name: Japanese oyster) is the mollusk most commonly consumed worldwide. Along with *Mytilus* spp. (common name: mussels), they have a global geographical distribution, facilitated by features such as high fertility, rapid growth, and resistance to environmental variations (salinity, temperature, etc.). These are euryhaline species, whose natural habitat is in the lower limit of the intertidal zone until the subtidal (about 15 m) in estuaries and coastal lagoons for *C. gigas*, and in the high intertidal to subtidal regions in estuarine areas to oceanic seawaters for *Mytilus* spp. [1,6]. On the other hand, *R. decussatus* (common name: clams) are mostly cultivated in Portugal, Spain, the Atlantic coast of France and in the Mediterranean basin. This species is usually found in shallow waters, burrowed in sand and silty mud. These bivalve species feed by filtration of phytoplankton and organic matter (detritus) from the surrounding water [1,7,8]. This type of feeding allows an accumulation of numerous contaminants in these animals, such as toxins, antibiotic residues, bacteria, viruses, and protozoa. Therefore, bivalve mollusks can suffer from numerous infectious diseases, especially if cultured in high densities, that cause high mortality rates and have a significant commercial impact [9]. Among the most frequent diseases are those caused by bacteria, which leads to an increase in antibiotic consumption to treat and prevent the spread of these diseases. Moreover, the accumulation of antibiotic residues can submit commensal and pathogenic bacteria of these organisms and bacteria from the aquatic environment to a selective pressure, contributing to the rise of antibiotic resistance [7]. Among the most frequently found bacteria in bivalve mollusks are those belonging to the *Proteobacteria* phylum [10,11]. In this phylum, we can find normal commensal bacteria (e.g., *Bacillus* spp., *Vibrio* spp. and *Aeromonas* spp.) and non-commensal bacteria (e.g., *Shewanella algae*). Bacteria from both groups can become pathogenic to these organisms [9,12]. Previous studies detected antibiotic resistance, namely to amoxicillin and quinolones, in bivalve mollusks [13,14]. Other studies estimate 700,000 deaths per year around the world due to antibiotic-resistant bacteria [15]. Antibiotic resistance is a growing and global threat, reaching not only human, but also veterinary medicine, since there are studies that indicate the transfer of antibiotic resistance genes between these two reservoirs [16,17]. Bacteria present in bivalve mollusks (e.g., *Vibrio* spp. and *Photobacterium damselae*) can be responsible for infections in humans through the consumption (e.g., gastroenteritis) or handling of these organisms (e.g., wound infections that can evolve to necrotizing fasciitis with multiple organ failure and septicemia) [18,19,20].

Given this scenario, we designed a study to understand the diversity of antibiotic-resistant bacterial species present in the three mainly produced bivalve mollusks in two locally distant regions of Portugal (*R. decussatus*, *Mytilus* spp. and *C. gigas*), and the molecular mechanisms of antibiotic resistance that are circulating in these aquaculture environments.

## 2. Results

Overall, after the initial screening with selective media containing antibiotics (amoxicillin, cefotaxime, chloramphenicol, colistin, nalidixic acid and/or oxytetracycline), two hundred and twenty-two bacterial strains were recovered from the bivalve mollusks’ samples included in this study. One hundred and ninety-two were Gram-negative bacteria, whereas only thirty were Gram-positive bacteria. Gram-negative bacteria prevail in all three species of bivalve mollusks, when compared with Gram-positive bacteria. All bacterial families and respective species found in this study are listed in Table 1.

### 2.1. Bacterial Diversity in Clams Samples

**Figure 1:**
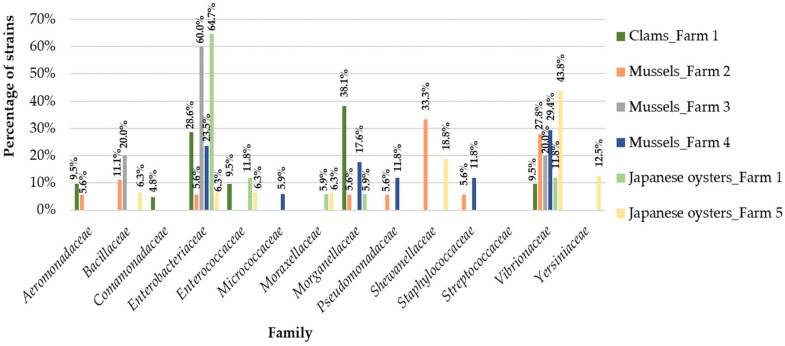
Distribution of the bacterial families among the five aquaculture farms in the summer.

**Figure 2:**
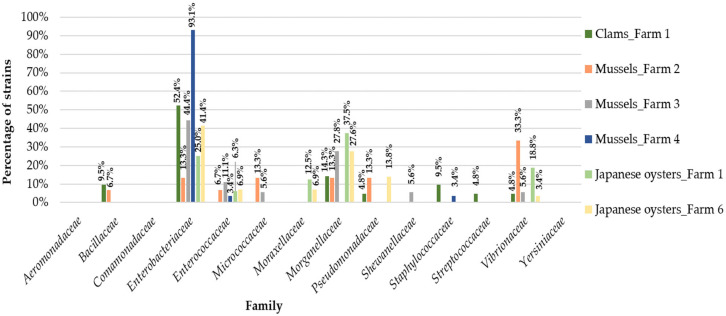
Distribution of the bacterial families among the five aquaculture farms in the autumn.

In clams samples we identified ten different families of bacteria (Figure 1 and Figure 2 and Table S1): *Aeromonadaceae*, *Comamonadaceae*, *Enterococcaceae* (only in summer), *Bacillaceae*, *Staphylococcaceae*, *Streptococcaceae* (only in autumn), *Enterobacteriaceae*, *Morganellaceae*, *Pseudomonadaceae* and *Vibrionaceae* (in both seasons). In summer, *Morganellaceae* was the most frequent bacterial family (38.1%). In fact, *Morganellaceae* appears more in this season in clams than in other bivalves studied (*p* = 0.02; Table 2). However, in autumn the results differ, with *Enterobacteriaceae* representing the most frequently isolated bacterial family (52.4%).

### 2.2. Bacterial Diversity in Mussels samples

Ten different families of bacteria were found among mussels samples: Aeromonadaceae (only in summer), Enterococcaceae (only in autumn), Bacillaceae, Enterobacteriaceae, Micrococcaceae, Morganellaceae, Pseudomonadaceae, Shewanellaceae, Staphylococcaceae and Vibrionaceae (in both seasons). In aquaculture farm 2, the most frequently found bacterial family in summer was Shewanellaceae (33.3%), whereas in autumn the most frequently found was Vibrionaceae (33.3%). In aquaculture farm 3, Enterobacteriaceae predominated in both seasons (with 60.0% in summer and 44.4% in autumn). In aquaculture farm 4, the most frequently found bacterial family in summer was Vibrionaceae (29.4%) and in the autumn it was Enterobacteriaceae (93.1%) (Figure 1 and Figure 2 and Table S1). Overall, we verified that Enterobacteriaceae appears more in autumn in mussels than in other bivalves (*p* ≤ 0.01) and was most frequent in autumn than in summer among mussels samples (*p* ≤ 0.01). Our statistical analyses showed that Morganellaceae were not usually associated with mussels samples (protective association; *p* = 0.02). In this bivalve species, Shewanellaceae and Vibrionaceae were most frequently found in summer than in autumn (*p* ≤ 0.01 and *p* = 0.02, respectively) (Table 2).

### 2.3. Bacterial Diversity in Japanese Oysters Samples

In Japanese oysters samples we identified nine different bacterial families: *Bacillaceae*, *Shewanellaceae*, *Yersiniaceae* (only in summer), *Pseudomonadaceae* (only in autumn), *Enterobacteriaceae*, *Enterococcaceae*, *Moraxellaceae*, *Morganellaceae* and *Vibrionaceae* (in both seasons). In this group of samples, in aquaculture farm 1 *Enterobacteriaceae* was the most frequently found bacterial family in summer (64.7%; Figure 1 and Figure 2 and Table S1). Indeed, this family appears more in summer in Japanese oysters than in the other bivalves studied (*p* ≤ 0.01; Table 2). In autumn, *Morganellaceae* was the most common family (37.5%) in aquaculture farm 1. In fact, *Morganellaceae* is more frequently found in autumn than in summer on all the studied aquaculture farms (*p* ≤ 0.01). In farm 5 (only samples collected in summer), *Vibrionaceae* was the most frequently found bacterial family (43.8%), while in farm 6 (only samples collected in autumn), *Enterobacteriaceae* predominated (41.4%).

### 2.4. Initial Evaluation of Decreased Susceptibilities

**Figure 3:**
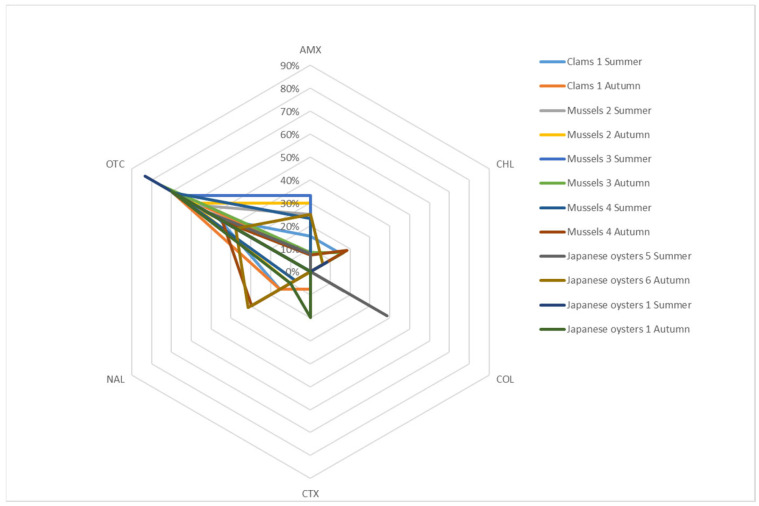
Decreased susceptibilities found in bivalves samples. These results were obtained through the initial screening with selective media containing antibiotics and do not include known intrinsic resistances. AMX: amoxicillin; CHL: chloramphenicol; COL: colistin; CTX: cefotaxime; NAL: nalidixic acid; OTC: oxytetracycline.

Initial screening with selective media containing antibiotics allowed the identification of decreased susceptibilities. Figure 3 (Figure S1 and Table S2) presents the results of this screening (eliminating known intrinsic resistances for the analysis). Decreased susceptibility to oxytetracycline prevails in all bivalve species, aquaculture farms and seasons.

In clams, no decreased susceptibility to colistin was found and decreased susceptibility to chloramphenicol was only identified in samples collected in summer (15.4%).

In mussels, no reduced susceptibility to cefotaxime was found. In contrast, amoxicillin and oxytetracycline reduced susceptibility were present in all farms and seasons. Autumn was the only season where reduced susceptibility to chloramphenicol was found (16.7% in farm 3 and 18.5% in farm 4). However, reduced susceptibility to colistin was only found in a sample from farm 2, collected in summer (16.7%). Reduced susceptibility to nalidixic acid was not found in farm 3.

Although, in Japanese oysters, only decreased susceptibility to oxytetracycline was found in all farms and seasons, this decreased susceptibility was not so frequent in these bivalve species (protective association) when compared to other species of bivalve analyzed in this study (*p* ≤ 0.01; Table 2). Furthermore, this decreased susceptibility appears more associated with summer than autumn in these bivalves (*p* = 0.02; Table 2). Decreased susceptibility to amoxicillin and colistin were only found in samples from region B (farms 5 and 6), whereas decreased susceptibility to cefotaxime was only recovered in samples from region A (farm 1). Decreased susceptibility to nalidixic acid was only observed in samples collected in autumn (31.3% in farm 6 and 10.0% in farm 1).

### 2.5. Antibiotic Susceptibility of β-Lactamase- and Plasmid-Mediated Quinolone Resistance (PMQR)-Producing Strains

Investigation of resistance genes by PCR revealed six *qnrA* genes among *S. algae*, ten *qnrB* genes among *C. braakii*, *C. freundii* and *E. coli*, and three *oqxAB* genes from *R. ornithinolytica* strains (Table 3). *qnrA* genes were found in region A and B aquaculture farms, in both seasons, although they predominated in summer. However, *qnrB* and *oqxAB* genes were only found in region A aquaculture farms in autumn. Three PMQR-producing strains (*C. braakii*, *C. freundii* and *S. algae*) revealed a susceptibility profile to all quinolones tested, with zone diameters ranging from 31 to 33 mm (disk diffusion) and concentrations of <0.015 to 0.125 mg/L (MIC) for ciprofloxacin, concentrations of 0.5 to 2 mg/L for flumequine, and a zone diameter of 27 mm for levofloxacin. The remaining sixteen PMQR-producing strains revealed a decreased susceptibility to at least one quinolone tested, with concentrations of 0.5 to >16 mg/L for ciprofloxacin and 2 to 64 mg/L for flumequine. Non-susceptibilities to amoxicillin, colistin and oxytetracycline (MIC concentrations of 8 to 64 mg/L for the last one) were also found among *S. algae* strains. All *R. ornithinolytica* harboring an *oqxAB* gene were also resistant to oxytetracycline, with concentrations ranging from 16 to 64 mg/L. Decreased susceptibility to β-lactam antibiotics among *Citrobacter* spp. was mostly intrinsic. This genus also revealed decreased susceptibility to phenicols and oxytetracycline, with a concentration of 32 mg/L for chloramphenicol (*C. braakii*), 8 to 16 mg/L for florfenicol and 8 mg/L for oxytetracycline. *E. coli* harboring *qnrB19* and *bla*_TEM-1_ genes revealed non-susceptibility to β-lactam antibiotics as well, in addition to non-susceptibilities to chloramphenicol, florfenicol, ciprofloxacin, flumequine, oxytetracycline and trimethoprim/sulfamethoxazole. Our study detected 11 multidrug resistance strains: one *C. braakii* recovered from clams, one *C. freundii* recovered from Japanese oysters, and eight *E. coli* and one *S. algae* recovered from mussels.

## 3. Discussion

As we observed in our study, bivalve mollusks usually concentrate a great diversity of bacterial species/families, which makes them susceptible to various diseases and may represent a risk to human health, since some of these bivalves are eaten raw (e.g., oysters). Indeed, statistically significant differences in bacterial composition between bivalve species from the same aquaculture farm and season (clams and Japanese oysters from farm 1, region A) were detected. Within the same bivalve species, we also observed variations between farms from the same region (mussels in region A) and different regions (Japanese oysters in region A and B), although these variations were not statistically significant. Fernández et al. detected variations in bacterial composition of post larvae and adult stages of *Crassostrea corteziensis*, *Crassostrea gigas* and *Crassostrea sikamea* at different cultivation sites [13]. They concluded that the most frequent phyla were *Proteobacteria*, *Bacteroidetes*, *Actinobacteria* and *Firmicutes* (in that order), using a high-throughput sequencing approach (pyrosequencing). In a different study, Pierce and Ward evaluated the gut microbiome from *Crassostrea virginica* and *Mytilus edulis* and confirmed that these species had similar (but not identical) gut microbiomes that vary with the seasons [11]. The most abundant phyla were *Proteobacteria*, *Tenericutes*, *Verrucomicrobia*, *Bacteroidetes*, *Cyanobacteria*, *Planctomycetes*, *Actinobacteria*, *Firmicutes*, and *Fusobacteria*. In our study, the most frequently identified phylum was also *Proteobacteria*, which comprised the following families: *Aeromonadaceae*, *Comamonadaceae*, *Enterobacteriaceae*, *Moraxellaceae*, *Pseudomonadaceae*, *Shewanellaceae*, *Vibrionaceae*, and *Yersiniaceae*. These findings agree with the hypothesis previously proposed that bacterial composition in bivalve mollusks is influenced by host species and respective life stage, diet, rearing conditions, bacterial composition of the aquatic habitat, salinity, and temperature [9,21,22].

The bacteria found in this study belonging to the genera *Acinetobacter*, *Aeromonas*, *Bacillus*, *Micrococcus*, *Photobacterium*, *Pseudomonas*, and *Vibrio* are ubiquitous in the water environments and commensal microbiota of bivalves [9]. Some of these bacteria, especially those belonging to *Proteobacteria* phylum, are important for bivalve mollusks’ metabolism, since they are able to fix nitrogen in the gastrointestinal tract of these organisms and degrade cellulose and agar (the main elements of the food ingested by bivalve mollusks) [21]. Species belonging to the genera *Aeromonas*, *Pseudomonas* and *Vibrio* are also responsible for diseases in bivalve larvae [9]. Information about the frequency and pathogenicity of other genera found in this study in bivalve mollusks is scarce. However, there are several studies reporting human infections caused by all seven genera described above, namely wound infections, foodborne diseases (by ingestion of raw seafood), myonecrosis, septicemia, necrotizing fasciitis, empyema, bacteremia, endocarditis, severe respiratory, urinary and biliary tract infections, meningitis, and keratitis [9,18,19,20,22,23,24,25,26,27].

In addition to commensal microbiota of bivalve mollusks, we also found non-commensal bacteria, some already reported in bivalve mollusks, others with no information about their presence in these organisms. The genera *Enterobacter*, *Enterococcus*, *Klebsiella*, *Proteus*, *Providencia,* and *Staphylococcus*, as well as the species from *Citrobacter freundii* complex, *Escherichia coli*, *Morganella morganii*, *Shewanella algae* and *Vagococcus fluvialis*, found in this study, were already reported in bivalve mollusks (especially clams, mussels and oysters) [12,13,28,29,30,31,32,33,34,35,36]. These groups of bacteria, some of which are fish pathogens, are commonly found in aquatic environments [12,25,34,35,37,38]. All these bacteria were already associated with human infections, such as ear and eye infections, osteomyelitis, infective arthritis, endocarditis, bacteremia, meningitis, intestinal and urinary tract infections, brain abscess, peritonitis, enteritis and septicemia, and some are recognized as important agents in nosocomial infections [12,13,34,37,39,40,41,42,43,44,45,46,47,48,49].

To our knowledge, this is the first report associating Comamonas aquatica, Escherichia fergusonii, Lactococcus garvieae, Moraxella osloensis, Pseudocitrobacter faecalis, Raoultella ornithinolytica, and Serratia marcescens with bivalve mollusks from aquaculture. These bacteria were already recovered from a wide range of environments, such as water, soil, plants, fish, insects, milk, cheese, sugar cane, mango, and the feces of warm-blooded animals, among others [45,50,51,52,53,54,55,56,57]. Furthermore, they are responsible for bacteremia, septic shock, biliary, gastrointestinal, urinary tract and wound infections, meningitis, infective endocarditis, lumbar osteomyelitis, and hepatic abscess in humans [52,55,56,57,58].

Non-commensal bacteria of bivalve mollusks such as Enterobacter spp., Enterococcus faecalis, Enterococcus faecium, E. coli, Klebsiella aerogenes, K. pneumoniae, M. osloensis, M. morganii, and Providencia rettgeri could be indicators of fecal contamination, since these bacteria are widely found in the gastrointestinal tract of humans and several other animals [13,25,32,34,35,40,46,48,51]. These and other bacteria can enter aquaculture farms through runoff from land (especially during periods of high precipitation), sewage, maritime traffic and birds or marine mammals. Fecal material from land and sewage can concentrate a high bacterial diversity, as well as several heavy metals, antibiotics, and organic substances, promoting a selective pressure on bacteria normally present in an aquatic environment [13].

The initial screening with selective media containing antibiotics and, subsequently, the MIC results revealed the prevalence of decreased susceptibility to oxytetracycline in all bivalve species, aquaculture farms and seasons. This prevalence could be explained by the high frequency of prescription of this antibiotic in aquaculture, due to broad spectrum of activity, low cost, and potency [7]. With one exception, we could not establish an association between an antibiotic and a specific bivalve species, location, or season. The exception was the decreased susceptibility to oxytetracycline that was statistically associated (*p* = 0.02) with summer in Japanese oysters.

Decreased susceptibility to β-lactams was also found in the present study. This antibiotic class is frequently used in aquaculture in several countries [25] and high resistance rates are usually observed in bivalve mollusks (especially regarding amoxicillin), often associated with *bla*_TEM_ and *bla*_CTX-M_ [13]. In our study, we also detected the *bla*_TEM-1_ gene in eight multidrug resistant *E. coli* strains with decreased susceptibility to amoxicillin, amoxicillin/clavulanic acid and/or ceftazidime. Noteworthy, strains with non-susceptibilities to β-lactams might have other resistance mechanisms not studied here (e.g., efflux pumps).

The other decreased susceptibility detected in this study was to chloramphenicol, although this antibiotic has already been banned for use in food-producing animals in Europe since the 1990s. This decreased susceptibility can persist in the environment due to coselection with other antibiotics (especially florfenicol, which is widely used in aquaculture, and decreased susceptibility to this antibiotic was also found in this study) and/or heavy metals. Moreover, there are soil bacteria that are capable of producing this substance [25]. Other studies also confirmed the presence of this antibiotic resistance in bivalve mollusks [13,59].

Worryingly, our study detected decreased susceptibility to colistin, an antibiotic of last resort against multidrug resistant Gram-negative bacteria in human medicine. This antibiotic is also used in veterinary medicine, including aquaculture, the latter hypothesized by some authors as the source of certain colistin resistance genes [60,61]. In our study, fourteen strains revealed decreased susceptibility to this antibiotic. Of these, seven, identified as *Shewanella algae* and *Photobacterium damselae*, had non-intrinsic resistance and were isolated from mussels collected in aquaculture farm 2 and Japanese oysters collected in aquaculture farm 5 (in the region A and B, respectively). These results may reflect a selective pressure in these regions that facilitates the dissemination of strains with decreased susceptibility to colistin. No plasmid-mediated colistin resistance-encoding genes were detected, which suggested that other resistance mechanisms not studied here are responsible for the decreased susceptibility to this antibiotic (other *mcr*-variant genes; efflux pumps; or *pmrC*, *pmrE*, *mgrB* genes, among others genes and operons that play a role in lipopolysaccharide modification and consequent decreased susceptibility to colistin) [62]. Previous studies had already detected decreased susceptibility to colistin and *mcr-1* gene in clams and mussels, respectively [61,63].

Our study revealed a low prevalence of decreased susceptibility to nalidixic acid (13%) in all bivalve samples analyzed. This antibiotic belongs to a class commonly used in aquaculture, quinolones [25]. Former studies in bivalve mollusks, also detected low levels of decreased susceptibility to quinolones [13,14]. Of the twenty strains with decreased susceptibility to nalidixic acid, only three strains of *R. ornithinolytica* revealed a positive result for the *oqxAB* gene. These three strains also revealed decreased susceptibility to other quinolones, such as flumequine and ciprofloxacin. Investigation of quinolones resistance genes by PCR in all Gram-negative strains, regardless their phenotype, revealed the presence of six *qnrA*-type genes among *S. algae* and ten *qnrB*-type genes among *C. braakii*, *C. freundii* and *E. coli*. Interestingly, not all strains harboring a *qnr* gene revealed a resistance phenotype to quinolones (one *C. braakii* with a *qnrB*-type, one *C. freundii* with a *qnrB44* and one *S. algae* with a *qnrA12*). This may be caused by non-functional proteins, such as in the case of *C. braakii* with a deletion in *qnrB* gene that originated a premature stop codon, or a low expression of these genes, difficult to detect by phenotypic methods [64]. These results highlight the importance of using both phenotypic and genotypic methods in research of antibiotic resistances/resistance genes, since there is not always phenotypic and/or genotypic expression. Although *qnrA*, *qnrB* and *oqxAB* genes are frequently found in aquaculture environments [25,65,66], little information is known about their frequency in bivalve mollusks, thus this study can contribute to better knowledge in this field.

All *E. coli* strains that harbor a *qnrB19* gene had a decreased susceptibility to quinolones (ciprofloxacin and/or flumequine) and also presented resistance to the combination trimethoprim/sulfamethoxazole (also used in aquaculture [25]). This resistance was previously reported in bivalve mollusks [67] and could be associated with the acquisition of genes *sulI* and *sulII* (for sulfamethoxazole resistance) and *dhfrI* and *dhfrII* (for trimethoprim resistance), causing the alteration of the antibiotic target [68].

In this study, we observed low resistance rates/few resistance genes to the antibiotics tested (except for oxytetracycline). However, it is important to implement surveillance plans in aquaculture farms, since this environment can be a reservoir of antibiotic resistance and/or antibiotic resistance genes. The implementation of measures that help to prevent outbreaks is also crucial, because fighting an outbreak is more difficult and expensive. Examples of such measures are limiting stock movements, avoiding exposure to elevated temperatures and high or low salinity, strict hygiene measures, and decreasing stock densities, among others. In the presence of an outbreak, it is important to identify the pathogen responsible and, if possible, to test its susceptibility to antibiotics, so that veterinarians can use a narrow-spectrum antibiotic at the correct concentration. Whenever possible, antibiotic administration by bath or feed should be avoided, giving preference to more individual methods to prevent the exposure of healthy individuals and the aquatic environment to a selective pressure. Investment in alternatives to antibiotics should be made, such as antimicrobial peptides (produced by several species of bivalve mollusks), bacteriophages, probiotics, and vaccines, always considering animal welfare and the product’s safety for human consumption [9,69].

## 4. Materials and Methods

### 4.1. Sample Characterization

The Portuguese Institute for the Sea and Atmosphere (IPMA) provided the bivalve samples used in this study. In summer of 2019, were collected one sample of clams (*R. decussatus*) and one sample of Japanese oyster (*C. gigas*) in aquaculture farm 1 from region A (south of Portugal); three samples of mussels (*Mytilus* spp.) from aquaculture farms 2, 3 and 4, also in region A; and one sample of Japanese oyster collected from aquaculture farm 5 in region B (central region of Portugal). The aquaculture farms from region A present in this study are distributed along its coastline.

In autumn of 2019, the sampling previously described was repeated for both regions, except for the sample of Japanese oyster in region B, which was collected in a different aquaculture (farm 6).

All samples were frozen and transported on ice to the National Institute of Health Dr. Ricardo Jorge, where they were analyzed. In this study, one sample corresponds to 3 to 10 individuals, depending on the species (minimum 50 g, for each sample).

### 4.2. Bacterial Isolation and Identification

Fifty grams of each sample were homogenized in peptone water (Stomacher 80 Biomaster^®^, Seward, UK), making a 1:10 dilution, and incubated for 12 to 18 h at 37 °C. Each dilution was plated in selective media, containing specific concentrations of different antibiotics (allowing an initial screening of decreased susceptibilities), and incubated for 18 to 20 h at 37 °C. Aeromonas agar, MacConkey agar and Thiosulfate Citrate Bile Salts Sucrose Agar contained the following standard antibiotic concentrations to select antibiotic resistant strains: 100 mg/L of amoxicillin, 2 mg/L of cefotaxime, 20 mg/L of chloramphenicol, 0.5 mg/L of colistin, 50 mg/L of nalidixic acid and 8 mg/L of oxytetracycline.

Mannitol salt agar and UriSelect™4 chromogenic agar contained 8 mg/L of oxytetracycline. Plates with and without antibiotic were used as controls. Colonies with different morphology (to avoid duplications) were selected and DNA extracted, according to manufacturer’s instructions (MagNA Pure 96 Instrument, Roche, Manheim, Germany). Strains were identified by MALDI-TOF and amplification of the 16S rRNA gene, as previously described [70].

### 4.3. Statistical Analyses of Results

Statistical analyses were performed to detect positive or negative associations between bivalve species and each bacterial family, bivalve species/bacterial family and season, *C. gigas*/bacterial family and location, bivalve species/bacterial family and nonsusceptibility to different classes of antibiotics (using the results from the initial screening in selective media). Only factors identified as statistically significant are shown. Fisher’s exact test was used to assess differences in bacterial families/season/location/nonsusceptibility to different classes of antibiotics between bivalve species and one-tailed *p* values of ≤0.05 were considered to be statistically significant. Associations were established by calculation of odds ratios with 95% confidence intervals. The null hypothesis was rejected for *p* values of ≤0.05. All statistical analyses were calculated using OpenEpi software, version 3.01 [71].

### 4.4. Molecular Detection of Resistance Genes

All Gram negative strains were investigated for the presence of *bla*_OXA-48_, *bla*_VIM_, *bla*_IMP-1-type_, *bla*_NDM_*, bla*_KPC_*, bla*_GES_, *bla*_SME_ (β-lactams resistance genes), *qnrA*, *qnrB*, *qnrC*, *qnrD*, *qnrS*, *aac(6′)-Ib*, *qepA* (quinolones resistance genes), *mcr-1*, *mcr-2*, *mcr-3*, *mcr-4 mcr-5* and *mcr-9* genes (colistin resistance genes) through Polymerase Chain Reaction (PCR), using primers already reported [25], with the exception of *mcr-9* primers. Primers and conditions for the search of *mcr-9* gene are here described for the first time (mcr9-F, 5′-TTCCCTTTGTTCTGGTTG-3′, and mcr9-R, 5′–GGATTATAGACGCTGGTG-3′; initial denaturation at 94 °C for 7 min, followed by 30 cycles of 94 °C for 45 s, 55.6 °C for 45 s and 72 °C for 1 min and 45 s with a final extension at 72 °C for 10 min). For 5 strains recovered from MacConkey agar with cefotaxime, we investigated the presence of *bla*_CTX-M_, *bla*_TEM_, *bla*_SHV_, and *bla*_OXA-1-type_ (β-lactams resistance genes) [25]. Furthermore, the presence of *oqxAB* gene (a quinolones resistance gene) was investigated for 20 strains recovered from Aeromonas/MacConkey agar with nalidixic acid [25]. Four more strains were searched for *bla*_TEM_, *bla*_SHV_, and *bla*_OXA-1-type_ genes according to the antibiotic susceptibility testing (see next subtitle): two with an intermediate phenotype to ceftazidime and the other two with positive results in disc combination test (DCT).

All *Staphylococcus* spp. were tested for the presence of *mecA*, *mecC*, *vanA*, *vanB* and *vanD* genes [25], whereas all *Enterococcus* spp. were studied for the presence of *vanA*, *vanB* and *vanD* genes.

### 4.5. Antibiotic Susceptibility Testing of Strains with Resistance Genes

Antibiotic susceptibility was studied by disk diffusion (Bio-Rad, Marnes-la-Coquette, France) and minimum inhibitory concentration (MIC) by in-house broth microdilution for nineteen strains that revealed the presence of resistance genes. Antibiotics tested and respective concentrations and breakpoints are listed in Table 4. The antibiogram was completed with disc combination test (DCT), double disc synergy test (DDST), faropenem (10 µg) and temocillin (30 µg) to search for extended-spectrum β-lactamase (ESBL), metallo-β-lactamase (MBL), AmpC cephalosporinases and carbapenemases, as already reported [25]. The strains were considered multidrug resistant if they presented resistance to three or more structurally unrelated antibiotics. EUCAST species-specific intrinsic resistances were considered (https://www.eucast.org/expert_rules_and_intrinsic_resistance/) (accessed on 13 April 2021).

## 5. Conclusions

In recent years, there has been an increase in studies on microbiota and antibiotic resistances/resistance genes present in aquaculture, mainly on fish. This study presents an important contribution to fill the gaps in the knowledge of bacterial diversity and antibiotic resistance mechanisms in bivalve mollusks. We could observe a great variety of bacterial species and antibiotic resistances among clams, mussels and Japanese oysters, seasons, and locations. This fact highlights the need to study and adapt the surveillance plans and measures to prevent the spread of antibiotic resistance to each specific location and animal species. Therefore, bivalve mollusks can play an important role in monitoring these aquaculture environments, since their filter feeding habits make them excellent indicators of environmental pollution [13].

## Figures and Tables

**Table 1:** Bacterial families and respective species found in our study.

Bacterial Family	Bacterial Species	Bivalve Species
***Aeromonadaceae***	*Aeromonas punctata*	*R. decussatus*
	*Aeromonas* sp.	*Mytilus* spp.
***Bacillaceae***	*Bacillus* sp.	*R. decussatus*
	*Bacillus cereus* group	*C. gigas, Mytilus* spp. and *R. decussatus*
***Comamonadaceae***	*Comamonas aquatica*	*R. decussatus*
***Enterobacteriaceae***	*Citrobacter werkmanii*	
	*Pseudocitrobacter faecalis*	
	*Enterobacter cancerogenus*	*Mytilus* spp.
	*Escherichia fergusonii*	*C. gigas*
	*Raoultella ornithinolytica*	*Mytilus* spp. and *R. decussatus*
	*Citrobacter braakii*	*C. gigas* and *R. decussatus*
	*Klebsiella aerogenes*	
	*Enterobacter* spp. (*E. hormaechei, E. kobei*)	*C. gigas* and *Mytilus* spp.
	*Klebsiella* spp. (*K. pneumoniae*, *K. oxytoca*)	
	*Citrobacter freundii*	*C. gigas, Mytilus* spp. and *R. decussatus*
	*Enterobacter cloacae*	
	*Escherichia coli*	
***Enterococcaceae***	*Enterococcus* spp. (*E. faecalis*, *E. hirae*)	
	*Enterococcus faecium*	*C. gigas*
	*Vagococcus fluvialis*	*Mytilus* spp.
***Micrococcaceae***	*Micrococcus luteus*	
***Moraxellaceae***	*Acinetobacter* spp. (*A. beijerinckii*, *A. junii*, *A. pittii*, *A. ursingii*)	*C. gigas*
	*Moraxella osloensis*	
***Morganellaceae***	*Morganella morganii*	*C. gigas* and *R. decussatus*
	*Proteus hauseri*	*Mytilus* spp.
	*Proteus vulgaris*	*C. gigas, Mytilus* spp. and *R. decussatus*
	*Providencia* spp. (*P*. *rettgeri, P.* *stuartii)*	
***Pseudomonadaceae***	*Pseudomonas mendocina*	*Mytilus* spp. and *R. decussatus*
	*Pseudomonas putida*	*C. gigas* and *Mytilus* spp.
***Shewanellaceae***	*Shewanella algae*	
***Staphylococcaceae***	*Staphylococcus pasteuri*	*Mytilus* spp. and *R. decussatus*
	*Staphylococcus warneri*	*Mytilus* spp.
	*Staphylococcus xylosus*	*R. decussatus*
***Streptococcaceae***	*Lactococcus garvieae*	
***Vibrionaceae***	*Photobacterium damselae*	*C. gigas* and *Mytilus* spp.
	*Vibrio alginolyticus*	*C. gigas, Mytilus* spp. and *R. decussatus*
	*Vibrio fluvialis*	*Mytilus* spp.
	*Vibrio* spp. (*V. furnissii*, *V. vulnificus*)	*R. decussatus*
***Yersiniaceae***	*Serratia marcescens*	*C. gigas*

**Table 2:** Odds ratio (OR) and 95% confidence intervals (CI) (*p* ≤ 0.05) from the analyses of positive and negative associations between bivalve species and each bacterial family, bivalve species/bacterial family and season, *C. gigas*/bacterial family and location, bivalve species/bacterial family and nonsusceptibility to different antibiotic’s class (using the results from the initial screening in selective media).

Bivalve’s Common Name	Bivalve Species	Bacterial Family	Season	Collection Site	Antibiotic	OR ^1^	95% CI	*p* Value
***Clams***	*R. decussatus*/All	*Morganellaceae*	Summer	All	NA	6.933	1.02–54.16	0.02
***Mussels***	*Mytilus* spp./All	*Enterobacteriaceae*	Summer	All	NA	0.1908 (P)	0.05897–0.597	≤0.01
	*Mytilus* spp./All	*Enterobacteriaceae*	Autumn	All	NA	5.242	1.675–16.96	≤0.01
	*Mytilus* spp.	*Enterobacteriaceae*	Summer	All	NA	0.1689 (P)	0.0584–0.4595	≤0.01
	*Mytilus* spp.	*Enterobacteriaceae*	Autumn	All	NA	5.92	2.176–17.12	≤0.01
	*Mytilus* spp.	*Morganellaceae*	All	All	NA	0.437 (P)	0.1842–0.983	0.02
	*Mytilus* spp.	*Shewanellaceae*	Summer	All	NA	10.76	1.202–503	≤0.01
	*Mytilus* spp.	*Vibrionaceae*	Summer	All	NA	3.54	1.058–12.75	0.02
***Japanese oysters***	*C. gigas*/All	*Enterobacteriaceae*	Summer	All	NA	9.429	2.263–45.46	≤0.01
	*C. gigas*	*Morganellaceae*	Summer	All	NA	0.0692 (P)	0.001588–0.5202	≤0.01
	*C. gigas*	*Morganellaceae*	Autumn	All	NA	14.45	1.922–629.7	≤0.01
	*C. gigas*	All	All	All	Oxytetracycline	0.4167 (P)	0.2293–0.7527	≤0.01
	*C. gigas*	All	Summer	All	Oxytetracycline	2.786	1.061–7.35	0.02

Only significant associations are presented: *p* values ≤ 0.05 and confidence limits excluding null values (0, 1, or [n]). ^1^ Odds Ratio. (P) indicates an OR value for a protective or negative association; otherwise, values should be interpreted as a positive association. CI: Confidence intervals. NA: not applicable.

**Table 3:** Phenotype and genotype profile of the nineteen β-lactamase- and PMQR-producing strains.

Bacterial Species	Farm (No. of Strains)	Bivalve Mollusk Species	Season	Decreased Susceptibility Profile	AR Genes
***C. braakii***	1 (n = 1)	*R. decussatus*	A	AMX, AMC, FOX, CHL, FLO, OTC	*qnrB-type* ^1^
***C. freundii***	1 (n = 1)	*C. gigas*	A	AMC, CAZ, FOX, FLO, OTC	*qnrB44*
***E. coli***	4 (n = 8)	*Mytilus* spp.	A	(AMX), AMC, (CAZ), (CIP), CHL, FLO, (FMQ), OTC, SXT	*qnrB19,* *bla*_TEM-1_
***R. ornithinolytica***	4 (n = 3)	*Mytilus* spp.	A	(CIP), FMQ, NAL, OTC	*oqxAB*
***S. algae***	2 (n = 3)	*Mytilus* spp.	S	(AMX), (FMQ), OTC	*qnrA3*
	2 (n = 1)	*Mytilus* spp.	S	CIP, FMQ, OTC	*qnrA11*
	5 (n = 1)	*C. gigas*	S	COL	*qnrA12*
	3 (n = 1)	*Mytilus* spp.	A	FMQ, OTC	*qnrA2*

^1^ This *qnrB* sequence contains a premature stop codon due to naturally occurring deletion (suggesting a non-functional protein), so it was not possible to assign an allele number (accession number GenBank MW183827). AMX: amoxicillin; AMC: amoxicillin/clavulanic acid; FOX: cefoxitin; CAZ: ceftazidime; CIP: ciprofloxacin; CHL: chloramphenicol; COL: colistin; FLO: florfenicol; FMQ: flumequine; NAL: nalidixic acid; OTC: oxytetracycline; SXT: trimethoprim/sulfamethoxazole. AR: antibiotic resistance. A: Autumn. S: summer. Variable presence of nonsusceptibility phenotype is indicated by parentheses.

**Table 4:** Antibiotics and respective concentrations and breakpoints used, by bacterial family.

Bacterial Family	Method	Antibiotics Tested (Concentration)	Breakpoints
***Enterobacteriaceae***	Disk diffusion	AMC (20 + 10 µg), AZT (30 µg), FEP (30 µg), CTX (5 µg), FOX (30 µg), CAZ (10 µg), ERT (10 µg), IMP (10 µg), MEM (10 µg), PTZ (36 µg), CIP (5 µg), SXT (25 µg), GEN (10 µg)	EUCAST ^1^
	MIC	CHL, FLO, OTC FMQ CIP	CLSI VET08 ^2^ CASFM VET 2019 ^3^ EUCAST
***Shewanellaceae***	Disk diffusion	AZT (30 µg), FEP (30 µg), CAZ (10 µg), IMP (10 µg), MEM (10 µg), PTZ (36 µg), CIP (5 µg), LEV (5 µg), AN (30 µg), GEN (10 µg), NET (10 µg), TMN (10 µg)	EUCAST ^4^
	MIC	CHL, FLO, OTC, CIP, FMQ	CLSI M100 ^4,5^

AMC: amoxicillin/clavulanic acid; AN: amikacin; AZT: aztreonam; CAZ: ceftazidime; CHL: chloramphenicol; CTX: cefotaxime; CIP: ciprofloxacin; ERT: ertapenem; FEP: cefepime; FLO: florfenicol; FMQ: flumequine; FOX: cefoxitin; GEN: gentamicin; IPM: imipenem; LEV: levofloxacin; MEM: meropenem; NET: netilmicin; OTC: oxytetracycline; PTZ: piperacillin/tazobactam; SXT: trimethoprim/sulfamethoxazole; TMN: tobramycin; EUCAST: European Committee on Antimicrobial Susceptibility Testing. CLSI: Clinical and Laboratory Standards Institute; CASFM VET: Comité de l’antibiogramme de la Société Française de Microbiologie Recommandations Vétérinaires. ^1^ https://www.eucast.org/clinical_breakpoints/ (accessed on 13 April 2021). ^2^ https://clsi.org/ (accessed on 13 April 2021). ^3^ https://www.sfm-microbiologie.org/2019/07/09/casfm-veterinaire-2019/ (accessed on 13 April 2021). ^4^ Breakpoints for *Shewanella* spp. were not available, therefore breakpoints from EUCAST and CLSI M100 for *Pseudomonas* spp. were used, as reported elsewhere [72]. ^5^ Breakpoints for CHL were used for FLO as well; breakpoints for CIP were used for FMQ; and breakpoints for tetracycline were used for OTC.

## Data Availability

Not applicable.

## Supplementary Materials

The following are available online at https://www.mdpi.com/article/10.3390/antibiotics10091135/s1, Figure S1: Decreased susceptibilities found in bivalves samples. These results were obtained through the initial screening with selective media containing antibiotics and do not include known intrinsic resistances. AMX: amoxicillin; CHL: chloramphenicol; COL: colistin; CTX: cefotaxime; NAL: nalidixic acid; OTC: oxytetracycline. Table S1: Distribution of bacterial families among the six aquaculture farms in summer and autumn. Table S2: Percentage of strains with decreased susceptibility to antibiotics used in the initial screening.
