# Supplementary material for: Assessing the Bacterial Community Composition of Bivalve Mollusks Collected in Aquaculture Farms and Respective Susceptibility to Antibiotics
Source: Antibiotics (Basel). 2021 Sep 20;10(9):1135. doi: 10.3390/antibiotics10091135 (PMC8468174; doi:10.3390/antibiotics10091135)
Supplement: Supplementary file 1 [file antibiotics-10-01135-s001.zip › antibiotics-1341484-supplementary.pdf]

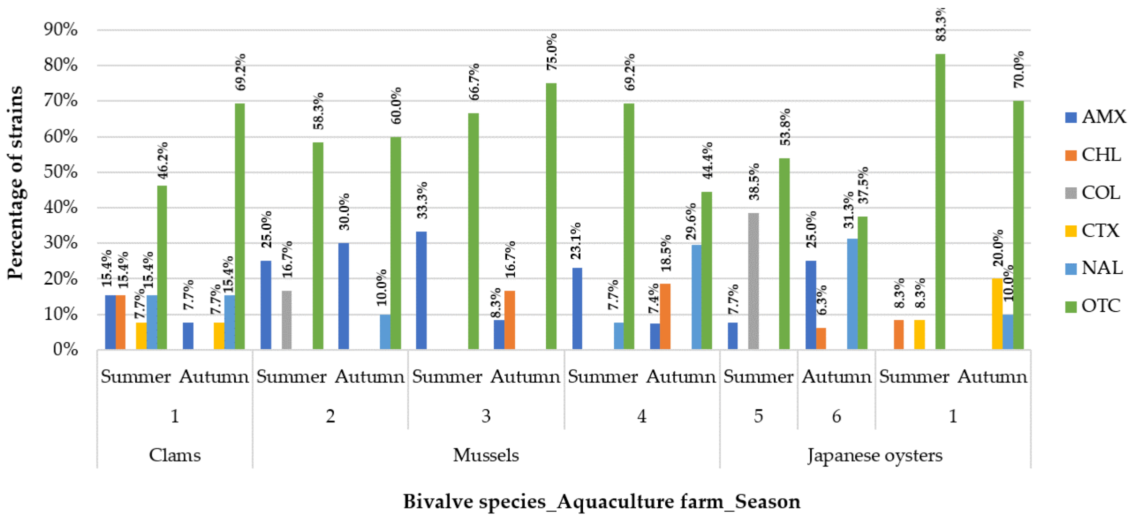

Figure S1. Decreased susceptibilities found in bivalves samples. These results were obtained through the initial screening with selective media containing antibiotics and do not include known intrinsic resistances. AMX: amoxicillin; CHL: chloramphenicol; COL: colistin; CTX: cefotaxime; NAL: nalidixic acid; OTC: oxytetracycline.

Table S1. Distribution of bacterial families among the six aquaculture farms in Summer and Autumn.

Family	Clams (n=42)		Mussels (n=102)						Japanese oysters (n=78)				Total
	Farm 1		Farm 2		Farm 3		Farm 4		Farm 1		Farm 5	Farm 6	
	Summer	Autumn	Summer	Autumn	Summer	Autumn	Summer	Autumn	Summer	Autumn	Summer	Autumn	
Aeromonadaceae	9.5%	0%	5.6%	0%	0%	0%	0%	0%	0%	0%	0%	0%	1.4%
Bacillaceae	0%	9.5%	11.1%	6.7%	20.0%	0%	0%	0%	0%	0%	6.3%	0%	3.2%
Comamonadaceae	4.8%	0%	0.0%	0%	0%	0%	0%	0%	0%	0%	0%	0%	0.5%
Enterobacteriaceae	28.6%	52.4%	5.6%	13.3%	60.0%	44.4%	23.5%	93.1%	64.7%	25.0%	6.3%	41.4%	40.5%
Enterococcaceae	9.5%	0%	0.0%	6.7%	0%	11.1%	0%	3.4%	11.8%	6.3%	6.3%	6.9%	5.4%
Micrococcaceae	0%	0%	0.0%	13.3%	0%	5.6%	5.9%	0%	0%	0%	0%	0%	1.8%
Moraxellaceae	0%	0%	0.0%	0%	0%	0%	0%	0.0%	5.9%	12.5%	6.3%	6.9%	2.7%
Morganellaceae	38.1%	14.3%	5.6%	13.3%	0%	27.8%	17.6%	0%	5.9%	37.5%	0%	27.6%	16.7%
Pseudomonadaceae	0%	4.8%	5.6%	13.3%	0%	0%	11.8%	0%	0%	0%	0%	13.8%	4.5%
Shewanellaceae	0%	0%	33.3%	0%	0%	5.6%	0%	0%	0%	0%	18.8%	0%	4.5%
Staphylococcaceae	0%	9.5%	5.6%	0%	0%	0%	11.8%	3.4%	0%	0%	0%	0%	2.7%
Streptococcaceae	0%	4.8%	0%	0%	0%	0%	0%	0%	0%	0%	0%	0%	0.5%
Vibrionaceae	9.5%	4.8%	27.8%	33.3%	20.0%	5.6%	29.4%	0%	11.8%	18.8%	43.8%	3.4%	14.9%
Yersiniaceae	0%	0%	0%	0%	0%	0%	0%	0%	0%	0%	12.5%	0%	0.9%
**Total**	100.0%	100.0%	100.0%	100.0%	100.0%	100.0%	100.0%	100.0%	100.0%	100.0%	100.0%	100.0%	100.0%

Table S2. Percentage of strains with decreased susceptibility to antibiotics used in the initial screening.

Bivalve species	Aquaculture farm	Season	Decreased susceptibility					
			AMX	CHL	COL	CTX	NAL	OTC
**Clams**	1	Summer	15.4%	15.4%	0%	7.7%	15.4%	46.2%
		Autumn	7.7%	0%	0%	7.7%	15.4%	69.2%
**Mussels**	2	Summer	25.0%	0%	16.7%	0%	0%	58.3%
		Autumn	30.0%	0%	0%	0%	10.0%	60.0%
	3	Summer	33.3%	0%	0%	0%	0%	66.7%
		Autumn	8.3%	16.7%	0%	0%	0%	75.0%
	4	Summer	23.1%	0%	0%	0%	7.7%	69.2%
		Autumn	7.4%	18.5%	0%	0%	29.6%	44.4%
**Japanese oysters**	5	Summer	7.7%	0%	38.5%	0%	0%	53.8%
	6	Autumn	25.0%	6.3%	0%	0%	31.3%	37.5%
	1	Summer	0%	8.3%	0%	8.3%	0%	83.3%
		Autumn	0%	0%	0%	20.0%	10.0%	70.0%
**Total**			13.6%	7.1%	4.5%	3.2%	13.0%	58.4%

These percentages do not include known intrinsic resistances. AMX: amoxicillin; CHL: chloramphenicol; COL: colistin; CTX: cefotaxime; NAL: nalidixic acid; OTC: oxytetracycline.
